# The “false-positive” conundrum: IgA reference level overestimates the seroprevalence of antibodies to SARS-CoV-2

**DOI:** 10.7189/jogh.11.05001

**Published:** 2021-01-16

**Authors:** Bruno Caramelli, Maria C Escalante-Rojas, Hiteshi K C Chauhan, Rinaldo F Siciliano, Marcio S Bittencourt, Antonio C Micelli

**Affiliations:** 1 InCor, University of São Paulo, São Paulo, Brazil; 2 Department of Cardiology, Fortis Hospital, Mohali, India; 3 Hospital Israelita Albert Einstein, Sao Paulo, Brazil; Faculdade Israelita de Ciências da Saúde Albert Einstein, São Paulo, Brazil; Center for Clinical and Epidemiological Research, University Hospital, University of Sao Paulo, Sao Paulo, Brazil; 4 Hospital das Clínicas, FMUSP, São Paulo, Brazil

## Abstract

**Background:**

On 12 June 2020, Brazil reached the second position worldwide in the number of COVID-19 cases. Authorities increased the number of tests performed, including the identification of antibodies to SARS-CoV-2 (IgG, IgA, and IgM). There was an overflooding of the market with several tests, and the presence of possible false-positive results became a challenge. The purpose of this study was to describe the seroprevalence and immunoglobulin blood levels in a group of asymptomatic individuals using the reference levels provided by the manufacturer.

**Methods:**

Levels of IgG and IgA antibodies to SARS-CoV-2 were determined in blood serum by the same ELISA (enzyme-linked immunoassay) test. Patients must be free of symptoms.

**Results:**

From 20 to 22 May 2020, 938 individuals were tested. There were 441 (47%) men, age 53 years (interquartile range (IQR) = 39-63.2). The sample included 335 (35.7%) subjects aged ≥60 years old. Subjects with a positive test were 54 (5.8%) for IgG and 96 (10.2%) for IgA and 42 (4.5%) for both IgG and IgA. The prevalence of IgG and IgA positive test was not different in men and women and not different in individuals under 60 and over 60 years of age. Conversely, analysing only individuals with positive tests, the levels of IgG in positive subjects were significantly higher than those with an IgA positive test, 3.00 (IQR = 1.68-5.65), and 1.95 (IQR = 1.40-3.38), respectively; *P* = 0.017. Additionally, individuals with isolated IgA positive tests had significantly lower levels of IgA than those with both IgA and IgG positive tests: 1.95 (IQR = 1.60-2.40) and 3.15 (IQR = 2.20-3.90), respectively, *P* = 0.005. These latter data suggest that IgA shows a deviation of the distribution to the left in comparison to IgG distribution data. Indeed, many subjects reported as IgA positive had immunoglobulin levels slightly elevated.

**Conclusions:**

In conclusion, we strongly suggest caution in the interpretation of IgA test results. This recommendation is more important for those with positive IgA just above the reference level.

The first case of severe acute respiratory syndrome coronavirus 2 (SARS-CoV-2) infection in Brazil was reported in São Paulo on 26 February 2020. On 12 March 2020, the first death related to the disease was confirmed, and on 13 March 2020, the first cases linked to community transmission were reported in Rio and São Paulo, the biggest cities in the Country. There was a rapid progression in the number of cases followed by subsequent requirement for intensive care support in proportions never experienced before. Exceptional efforts were made by the health authorities to cope with this monumental escalation of a snowballing pandemic. However, the initiatives and strategies deployed were not uniform across a Country with continental dimensions [1-5].

Despite strong recommendations, social distancing rates remained below desirable levels for most of the time period. Two possible consequences of the aforementioned were: a) intensive care units’ occupancy remained below 80%, most of the time and in majority of the cities of the Country, especially São Paulo and b) the number of new cases and the number of deaths reached either a stable plateau or showed a worrisome growth in some regions [6].

On 1 June 2020, in São Paulo, health authorities implemented a stepwise system to authorize economic activities to resume [7]. The main criteria included the average rate of intensive care units designated exclusively for SARS-CoV-2 patients, the number of new hospital admissions, and the death rate. On 12 June 2020, Brazil reached the dubious and worrisome distinction of attaining the second position Worldwide in the number of COVID-19 cases. In response, authorities, individuals on own initiative or requested by employers, clubs or airlines, increased the number of tests performed, both for the diagnosis of SARS-CoV-2 infection (molecular or PCR test) and the diagnosis of past infection in symptomatic and asymptomatic individuals through the identification of antibodies to SARS-CoV-2, ie, the determination of seroprevalence of IgG, IgA, and IgM [8-10].

Historically, seroprevalence data offers valuable information related to the development and progression of an epidemic outbreak [11]. However, the unique and largely unknown immunological response to SARS-CoV-2 limits the full epidemiological and clinical use of the test results [12-15]. Moreover, the determination of seroprevalence has exposed several caveats related to the use of qualitative instead of quantitative tests, the sum of different immunoglobin classes as a unique seroprevalence, and the presence of false-negative and false-positive results. Adding fuel to this uncertainty is the overflooding of the market with several tests, which has led to the unfortunate sequalae of pitting caregivers and patients on opposite ends of a divisive spectrum, arguing about test results that probably still require reference data. In our clinical practice, it has become increasingly common to field requests for an interpretation of serology testing from patients and friends. We observed that IgA results are more frequently positive, even in the absence of past clinical symptoms.

To test our hypothesis, we sought to analyze data obtained, at the end of May 2020, from a convenience sample of asymptomatic individuals analyzed by the same commercial diagnostic laboratory test to estimate the seroprevalence by age and gender, using the reference levels provided by the manufacturer.

## METHODS

This is a time-sensitive cross-sectional study that involved members from an upper-class social and sports club in São Paulo, Brazil. Blood samples were collected from May 20-22, 2020, in the Club's garage, a vast open space that allowed social distancing. Levels of IgG and IgA antibodies to SARS-CoV-2 were determined in blood serum by ELISA technology (Anti-SARS-CoV-2 IgG and IgA, EUROIMMUN BRASIL MEDICINA DIAGNOSTICA LTDA), and were performed in the same laboratory [7]. The cutoff level provided by the manufacturer for a positive test was an index greater than 1.1 for both IgG and IgA antibodies. Patients with fever, dyspnea, cough, or other symptoms related to active disease were not authorized by the club board to come and are not represented in this group. Demographic data was obtained from all patients.

Descriptive data are presented as absolute values, percentages, median, and interval interquartile range (IQR). For the comparisons we assumed non normal distribution and used nonparametric tests: Mann-Whitney test (Tabular results, Unpaired) for continuous variables, Pearson χ^2^ test with Yates' continuity for categorical variables and odds-ratio (OR), with 95% confidence interval (CI), calculated by Baptista-Pike method and performed using GraphPad Prism version 8.4.3 for Windows (GraphPad Software, San Diego, California USA, www.graphpad.com). Frequency distribution of different IgA and IgG levels was also analyzed and compared.

## RESULTS

**Figure 1:**
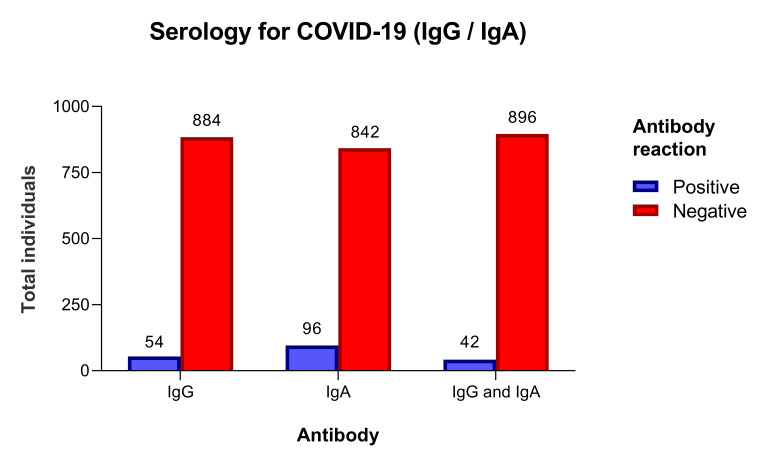
Distribution of total subjects by antibody, in the cohort of 938 individuals.

From May 20 to 22, 938 individuals were tested. There were 441 (47%) men and 497 women, age 53 years (IQR = 39.0-63.2). The sample included 335 (35.7%) individuals aged 60 or more years old. Individuals with a positive test (index ≥1.1) were 54 (5.8%) for IgG and 96 (10.2%) for IgA, and 42 (4.5%) for both IgG and IgA (**Figure 1**).

The prevalence of positive tests by age and gender groups, IgG, and IgA levels are depicted in **Table 1** and **Table 2**. The prevalence of IgG and IgA positive test was not different in men and women (OR = 1.14, 95% CI = 0.65-1.97for IgG and OR = 1.04, 95% CI = 0.68-1.59 for IgA; *P* = 0.937) and not different in individuals under 60 and ≥ 60 years old (OR = 1.12, 95% CI = 0.63-2.00, for IgG and OR = 0.87, 95% CI = 0.57-1.35; *P* = 0.619). Of note, the median IgG and IgA levels were nearly10-fold greater in individuals with a positive than in those with negative test (**Table 1** and **Table 2****)**.

**Table 1:** Demographic characteristics in IgG positive and negative subjects

	All IgG, n = 938, n (%)	IgG+, n = 54, n (%)	IgG -, n = 884, n (%)	OR (95%CI)	*P*-value
**Gender (n, %):**					
Male	441 (47.01%)	27 (50%)	414 (46.83%)	1.14 (0.65-1.97)	0.755
Female	497 (52.99%)	27 (50%)	470 (53.17%)		
**Age (n, %)**					
Age <60	603 (64.29%)	36 (66.67%)	567 (64.14%)	1.12 (0.63-2.00)	0.818
Age ≥60	335 (35.71%)	18 (33.33%)	317 (35.86%)		
**Age, median** (**IQR**)	53 (39.00-63.25)	48.5 (32.75-66.50)	53 (39.00-63.00)		0.563
**Ig level, median (IQR)**	0.30 (0.20-0.40)	3 (1.68-5.65)	0.30 (0.20-0.30)		<0.001

OR – odds ratio, CI – confidence interval, IQR – interquartile range

**Table 2:** Demographic characteristics in IgA positive and negative subjects*

	All IgA, n = 938, n (%)	IgA+, n = 96, n (%)	IgA-, n = 842, n (%)	OR (95% CI)	*P*-value
**Gender (n, %):**					
Male	441 (47.01%)	46 (47.92%)	395 (46.91%)	1.04 (0.68-1.59)	0.937
Female	497 (52.99%)	50 (52.08%)	447 (53.09%)		
**Age (years; n, %)**					
Age <60	603 (64.29%)	59 (61.46%)	544 (64.61%)	0.87 (0.57-1.35)	0.619
Age ≥60	335 (35.71%)	37 (38.54%)	298 (35.39%)		
**Age, median (IQR)**	53 (39.00-63.25)	52.50 (38.25-64.00)	53 (39.00-63.00)		0.688
**Ig level, median (IQR)**	0.30 (0.20-0.50)	1.95 (1.40-3.38)	0.20 (0.20-0.40)		<0.001

OR – odds ratio, CI – confidence interval, IQR – interquartile range

**Figure 2:**
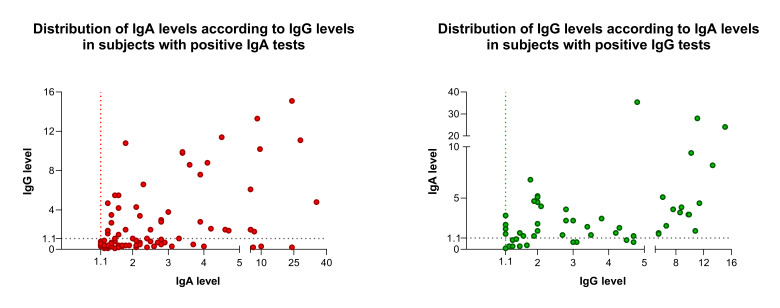
Frequency distribution of positive subjects by antibody IgG and IgA.

The frequency distribution of different IgA and IgG levels is shown in **Figure 2**. Remarkably, visual analysis of IgA demonstrates an apparent deviation of the distribution to the left in comparison to IgG distribution data. Conversely, analyzing only individuals with positive tests, the levels of IgG in individual with a positive test were significantly higher than in those with an IgA positive test, 3 (IQR = 1.68-5.65) vs 1.95 (IQR = 1.40-3.38). Additionally, individuals with isolated IgA positive test showed significant lower levels of IgA than those with both IgA and IgG positive tests: 1.95 (1.60-2.40) vs 3.15 (2.20-3.90).

## DISCUSSION

We report a cross-sectional study of qualitative (seroprevalence) and quantitative (serum levels of IgA and IgG) antibodies to SARS-CoV-2 in a cohort of 938 free-of-symptoms subjects in São Paulo, Brazil, at the end of May 2020. Previous reports in the literature privileged the analyzes & interpretation of seroprevalence (qualitative aspects) and longitudinal follow-up data [13-16]. The quality and strength of the information provided by antibody tests has been strongly criticized [17].

The present study was elaborated as a response to recurring clinical practice dilemma, ie, frequent requests for interpretation of results of serological tests by patients with positive results, especially for IgA. Considering the reference levels provided by the manufacturer, seroprevalence results were 5.8 and 10.2 for IgG and IgA, respectively. In our cohort, seroprevalence was not different in men and women but was higher in subjects less than 60 years old. This finding is similar to a recent publication from the United States. [18]

On the other hand, the quantitative data showed that the frequency distribution of Ig levels in positive subjects with positive results is quite different between IgG and IgA. Notably, IgA levels distribution in subjects with a positive test deviates to the left in comparison to IgG distribution data. This phenomenon indicates that many subjects reported as IgA positive have immunoglobulin levels just above the 1.1 reference level. The explanation for this finding is not provided by this or previous studies, but it is not possible to exclude the presence of false-positive results or a misleading reference value. The finding of higher immunoglobulin levels in subjects tested positive for IgG than those for IgA and the lower IgA levels in subjects tested positive only for IgA than those positive for both IgA and IgG also suggest a false-positive result or a recent infection. Positive results always create worries and a tense vibe for patients and their families. Health care systems and health authorities may get overburdened with strategizing as to the best policy towards these asymptomatic individuals.

To the present moment, there is no precise information regarding the clinical or epidemiological relevance of the almost 2-fold increase in the IgA in comparison to IgG levels. Considering the finding of this study, ie, that many subjects reported as IgA positive have immunoglobulin levels slightly elevated, we strongly suggest caution in the interpretation of IgA test results. This recommendation is more important for those with positive IgA just above the reference level. These individuals should go in for a repeat comprehensive evaluation and health care providers should not base their decision on this analysis. Confirmation of the hypothesis generated by these data needs future studies and could allow additional interpretation of them.
